# Probiotics and probiotic-based vaccines: A novel approach for improving vaccine efficacy

**DOI:** 10.3389/fmed.2022.940454

**Published:** 2022-10-13

**Authors:** Nesa Kazemifard, Abolfazl Dehkohneh, Shaghayegh Baradaran Ghavami

**Affiliations:** ^1^Basic and Molecular Epidemiology of Gastrointestinal Disorders Research Center, Research Institute for Gastroenterology and Liver Diseases, Shahid Beheshti University of Medical Sciences, Tehran, Iran; ^2^Department 4 – Materials and the Environment, Bundesanstalt für Materialforschung und -prüfung (BAM), Berlin, Germany; ^3^Department of Biology Chemistry Pharmacy, Freie Universität Berlin, Berlin, Germany

**Keywords:** probiotics, vaccine, vaccine efficacy, probiotic-based vaccines, gut microbiota, adaptive immunity

## Abstract

Vaccination is defined as the stimulation and development of the adaptive immune system by administering specific antigens. Vaccines' efficacy, in inducing immunity, varies in different societies due to economic, social, and biological conditions. One of the influential biological factors is gut microbiota. Cross-talks between gut bacteria and the host immune system are initiated at birth during microbial colonization and directly control the immune responses and protection against pathogen colonization. Imbalances in the gut microbiota composition, termed dysbiosis, can trigger several immune disorders through the activity of the adaptive immune system and impair the adequate response to the vaccination. The bacteria used in probiotics are often members of the gut microbiota, which have health benefits for the host. Probiotics are generally consumed as a component of fermented foods, affect both innate and acquired immune systems, and decrease infections. This review aimed to discuss the gut microbiota's role in regulating immune responses to vaccination and how probiotics can help induce immune responses against pathogens. Finally, probiotic-based oral vaccines and their efficacy have been discussed.

## Introduction

Vaccination is defined as the stimulation and development of the adaptive immune system by the administration of specific antigens. Vaccines help prevent and eradicate the mortality and morbidity of numerous infectious diseases (1). Vaccine efficacy (VE) is described as the incidence proportion between the vaccinated and non-vaccinated populations (2) and varies in different societies based on economic, social, and biological conditions (3, 4). Several suggested economic and social determinants, such as country income status, living conditions and access to healthcare appear to act indirectly and non-specifically on VE. In contrast, many but not all biological factors, such as co-infections, malnutrition, and enteropathy, presumably, act directly and proximally on VE (5). Gut microbiota also plays a crucial role in the development and regulation of the immune system; hence, its composition might affect how individuals respond to vaccinations (6, 7).

Gut microbiota develops alongside host development and is affected by genetics and environmental factors, and is an integral part of the human body (8, 9). The microbiota interacts with the host in many ways. Cross-talks between gut bacteria and the host immune system are initiated at birth during microbial colonization (10). This interaction promotes the intestinal epithelial barrier, immune homeostasis, protects from pathogen colonization (11), and inhibits deleterious inflammatory reactions that would harm both the host and its gut microbiota (12). Gut lymph nodes, lamina propria, and epithelial cells (mucosal immune system) form a protective barrier for the integrity of the intestinal tract (13). Therefore the gut microbiota composition can affect the normal mucosal immune system (14).

During gut microbiota development, especially in early life, various factors can affect and alter its composition. For instance, the human gut changes considerably during the first 2 years of life as children grow from breast milk-dominated diets to solid foods and are exposed to vast numbers of bacterial species (15). Therefore undernourished children have been reported to have less mature gut microbiota compared to healthy children (16). Diet serves as a significant factor in gut microbiota composition in adults too. Various studies reported that a higher-fat diet in healthy adults appeared to be associated with unfavorable changes in gut microbiota, fecal metabolomics profiles, and plasma pro inflammatory factors, which might result in long-term adverse consequences for health (17–19). In addition, metabolic diseases such as diabetes can alter the gut microbiome and disrupt gut bacterial equilibrium (20). Other factors, including physical activity, mental health, and obesity may also affect gut microbiota composition (21–23).

Imbalances in the gut microbiota composition, termed dysbiosis, can trigger several immune disorders through the activity of the adaptive immune system (24). For example, recent studies on this subject reported that germ-free (GF) mice had a reduced number of Th1 and Th17 cells. Th17 cells, which are grouped as CD4+ effector T cells that secrete IL-17, play an important role in host defense against extracellular pathogens and the development of autoimmune diseases (25–27). Moreover, in dysbiotic gut microbiota, the number of inducible Foxp3 Helio-Tregs (iTregs) is reduced significantly in colonic lamina propria (28). Other studies indicate that excessive use of antibiotics disrupting gut microbiota hemostasis in young children might delay or impair the proper development of IgG response and immune memory that profoundly impacts adulthood (29). This review highlighted studies about the relationship between gut microbiota and related immune responses after vaccination and the impact of gut microbiota dysbiosis on VE.

## Gut microbiota and vaccine efficacy

Cross-talk between the gut microbiome and the immune system by producing various metabolites and antimicrobial peptides directly regulates innate and adaptive immunity (30) and its failure to regulate inflammatory responses could increase the risk of developing inflammatory conditions in the gastrointestinal tract (31). Therefore the gut microbiota impacts the efficacy of various immune system-related interventions, including prevention of human immunodeficiency virus (HIV) infection (32, 33), cancer immunotherapy (34–36), and dysregulation in gut microbial composition associated with autoantibodies production and autoimmune diseases (37–40). Several studies were designed to evaluate the relationship between gut microbiota and immune responses to assess vaccine efficiency. A study by Pulendran et al. showed that antibiotic consumption resulted in a 10,000-fold reduction in gut bacterial composition and reduced specific neutralization and binding antibody responses against the influenza vaccine, and a significant association between bacterial species and metabolic phenotypes in the gut was displayed in this study (41). Furthermore, infants who received oral polio vaccine (OPV), intramuscular tetanus-hepatitis B, and intradermal Bacillus Calmette–Guérin (BCG) vaccines had detectable levels of *Bifidobacterium longum* (*B. longum*) and displayed higher specific T cell responses, serum IgG and fecal polio-specific IgA levels. In contrast, a higher relative abundance of *Enterobacteriales* and *Pseudomonadales* was associated with lower specific T cell responses and serum IgG levels (6, 42). Another study on infants receiving BCG, OPV, tetanus toxoid (TT), and hepatitis B virus confirmed the previous results that *Bifidobacterium* abundance in early infancy might increase the protective effects of vaccines by enhancing immunologic memory (7). The concurrent presence of non-polio enterovirus (NPEV) and oral polio vaccination can affect VE and reduce OPV seroconversion (43).

One of the critical factors in VE is the expression of Toll-like receptor 5 (TLR5) within 3 days after vaccination, which correlates to the amount of hemagglutination inhibition (HAI) titers 4 weeks after influenza vaccination (44, 45). TLR5 is a cell receptor for the recognition of flagellin and stimulates inflammatory signaling and immune responses (46). In addition, trivalent inactivated influenza vaccination of *Trl5–/–* mice resulted in reduced antibody titers. TLR5-mediated sensing of the microbiota also impacted antibody responses to the inactivated polio vaccine (47). NOD2 (Nucleotide-binding oligomerization domain 2), an intracellular pathogen recognition sensor, is associated with the immune system and VE stimulation (48, 49). Recognition of symbiotic bacteria by NOD2 in CD11c-expressing phagocytes helps the mucosal adjuvant activity of cholera toxin (CT), as confirmed by a study on mice (50).

One of the most influential factors that lead to dysregulation of gut microbiota dysbiosis is antibiotic exposure (51). In 1 study, it is demonstrated that antibiotics-induced dysbiosis in infant mice (but not adults) leads to impaired antibody responses and promotes *ex vivo* cytokine recall responses (52). Antibiotic-treated mice models also showed impaired oral immunization in response to cholera toxin (53) and dysregulation in the generation of anti-viral macrophages, virus-specific CD4 and CD8 T cells, and antibody responses following respiratory influenza virus infection (54, 55). Gut dysbiosis induced by antibiotics significantly decreased the activation of CD4+ T cells and CD8+ T cells and declined the level of memory of CD4+ T cells and CD8+ T cells in secondary lymphoid organs of the vaccinated animals (56). In a study on human adults with impaired microbiome induced by antibiotics, reduced antibody response to TIV in subjects with low pre-existing immunity to influenza virus was observed (41). However, adults receiving Rotavirus (RV), Pneumo23, and TT vaccines with antibiotics consumption showed increased fecal shedding of RV and changes in gut bacteria beta diversity which is associated with RV vaccine immunogenicity boosting (57). Although antibiotics consumption could not improve the immunogenicity of OPV in human infants, the reduction of enteropathy and pathogenic intestinal bacteria biomarkers were reported (58).

The composition of gut microbiota and its diversity are associated with the response of the immune system to vaccines. In this case, a study on specific pathogen-free layer chickens (SPF) showed that shifts in gut microbiota composition might result in changes in cell- and antibody-mediated immune responses to vaccination against influenza viruses (59, 60). Other experiments on adults receiving an HIV vaccine showed the immunogenicity of the vaccine was correlated with microbiota clusters (61). On the contrary, another study on human adults reported no differences in overall gut microbiota community diversity between humoral responders and non-responders to the oral *Salmonella Typhi* vaccine (62). Co-infection with porcine reproductive and respiratory syndrome virus (PRRSV) and porcine circovirus type 2 (PCV2) in pig models revealed that high growth outcomes were associated with several gut microbiome characteristics, such as increased bacterial diversity, increased relative abundance of *Bacteroides pectinophilus*, decreased *Mycoplasmataceae* species diversity, higher *Firmicutes:Bacteroidetes* ratios, increased relative abundance of the phylum *Spirochaetes*, reduced relative abundance of the family *Lachnospiraceae*, and increased *Lachnospiraceae* species (63). Diet is also influential on the gut microbiome and vaccine efficacy. A study showed that a gluten-free diet was associated with a reduced anti-tetanus IgG response, and it increased the relative abundance of the anti-inflammatory *Bifidobacterium* in the mice model (64).

Humans harbor several latent viruses, including cytomegalovirus (CMV) implicated in the modulation of host immunity (65). However, there is an insufficient understanding of the influence of lifelong persistent latent viral infections on the immune system (66). In a rhesus macaques model, subclinical CMV infection increased butyrate-producing bacteria and lower antibody responses to influenza vaccination (67).

Oral RV vaccines have the potential role in reducing the morbidity and mortality of RV infection that causes diarrhea-related death in children worldwide, but RV vaccines showed significantly lower efficacy in low-income countries (68, 69). A comparison between infants in India and Malawi and infants born in the UK showed that ORV immune response was significantly impaired among infants in the former. This result is linked with their gut microbiome composition, in which microbiota diversity was significantly higher among Malawian infants, while Indian infants had high *Bifidobacterium* abundance (70). Despite low RVV immunogenicity which was also reported in rural Zimbabwean infants, it was not associated with the composition or function of the early-life gut microbiome (71). Human gut microbiota transplanted pig models vaccinated with attenuated RVV showed significantly enhanced IFN-γ producing T cell responses and reduced regulatory T cells and cytokine production (72). Moreover, poor diet decreased total Ig and HRV-specific IgG and IgA antibody titers in serum or ileum and it increased fecal virus shedding titers in human infant microbiome transplanted pig models (57, 73, 74). In a study on rural Ghana's infants, RVV response was associated with an increased relative abundance of *Streptococcus gallolyticus*, decreased relative abundance of phylum *Bacteroidetes* and higher *Enterobacteria/Bacteroides* ratio (75). Another study reported that RVV response correlates with a higher relative abundance of bacteria belonging to Clostridium cluster XI and *Proteobacteria* (76). *Bacteroides thetaiotaomicron* is also associated with anti-rotavirus IgA titer (77). However, a study on Nicaraguan Infants reported a limited impact of gut microbial taxa on response to oral RVV (78).

Recent studies indicated that dysbiosis might be relevant in systemic severe acute respiratory syndrome coronavirus 2 (SARS-CoV-2) infections. Khan et al. indicated an association between dysbiosis and severe inflammatory response in coronavirus disease 2019 (COVID-19) patients. Decreased *Firmicutes*/*Bacteroidetes* ratio, induced by the depletion of *Faecalibacterium prausnitzii (*F. *prausnitzii), Bacteroides plebeius (B. plebeius)*, and *Prevotella*, which utilize fiber, and a relative increase in *Bacteroidetes* species is associated with raised serum IL-21 levels and better prognosis (79). A study on a cohort of 100 patients revealed that the composition of the gut microbiome in patients with COVID-19 correlates with disease severity, plasma concentrations of several inflammatory cytokines, and tissue-damaged associated chemokines. Patients with COVID-19 are recommended to consume beneficial microorganisms with immunomodulatory potentials, such as F. *prausnitzii, Eubacterium rectale*, and several *Bifidobacterium* species, and the dysbiosis persisted after the clearance of the virus (80, 81). Currently, controlling and preventing the spread of SARS-CoV2 infection is one of the critical challenges in the healthcare system. Vaccination is the best strategy to overcome this challenge (82). Among all recently proposed vaccines, an important note is to balance the humoral (neutralizing antibody) and T cell responses (83). Mucosal immunity is the most influential factor in preventing viral respiratory infections and response to vaccination. In this regard, the intestinal immune system is as important as the respiratory system's mucosal immunity (84). Thus, the intestinal immune system might be a promising approach for improving current SARS-CoV2 vaccination strategies (85). On the other hand, risk factors that reduce the immune system's defenses against SARS-CoV-2 infections could also reduce their responses to vaccination and increase vaccination's adverse effects. Thus gut dysbiosis is one of the mechanisms that can cause a pathological and impaired immune response to SARS-CoV-2 vaccination (86).

So far, most studies around vaccine efficacy and gut microbiota composition demonstrated that gut microbiota can influence vaccines' immunogenicity and the mucosal and acquired immunity against pathogens.

## The effects of probiotics on vaccine efficacy

Probiotics are live commensal microorganisms that have positive benefits for the host that are generally consumed as a component of fermented foods. They have an impact on both innate and adaptive immune systems and decrease infections (87, 88). A meta-analysis comprising 1,979 adults showed that probiotics and prebiotics effectively promote immunogenicity by influencing seroconversion and seroprotection rates in adults vaccinated with influenza vaccines (89).

*Bifidobacteria* (BIF) is one of the probiotics and beneficial bacteria for human and animal health, having roles in the prevention of infection, modulation of lipid metabolism, and reduction of allergic symptoms by stimulating the host's mucosal immune system and systemic immune response (90, 91). Consumption of *B*. *longum* BB536 in newborns showed an increase in the number of interferon-γ (IFN-γ), a representative cytokine for T helper 1 response, secretion cells, and the ratio of IFN-γ/IL-4 secretion cells (92). In addition, a combination of *B*. *longum* BL999 and *Lactobacillus rhamnosus* (*L*. *rhamnosus*) [LPR (CGMCC1.3724)] consumption after Hepatitis B vaccination resulted in improved antibody responses (93). The results of a study on adults who received seasonal influenza vaccines was the same. Probiotic consumption (*B*. *longum* bv. *infantis* CCUG 52,486, combined with a prebiotic gluco-oligosaccharide) could improve total antibody titers and seroprotection (94). *Bifidobacterium lactis* BB-12 and *Lactobacillus paracasei* (*L*. *paracasei*) 431 improved specific Antibody titers and seroconversion rates after influenza vaccination but there was no difference in INF-γ, IL-2, and IL-10 levels (95). In a randomized placebo-controlled, double-blinded prospective trial, the effect of probiotics [*Bifidobacterium bifidum, B. infantis, B. longum*, and *Lactobacillus acidophilus* (*L. acidophilus*)] on vaccination efficacy could not be proven statistically (96).

Strains of *Lactobacillus* are a subdominant component of the commensal human intestinal microbiota and are identified as a potential driving force in the development of the human immune system (97). They exert early immunostimulatory effects that may be directly linked to the initial inflammation responses in human macrophages (98). Chickens who received *Lactobacillus* spp as probiotics showed an increased major histocompatibility complex (MHC) II expression on macrophages and B cells. The number of CD4 + CD25 + T regulatory cells was also reduced in the spleen (99). In a study, the probiotic function of *Lactobacillus plantarum* (*L. plantarum*) was assessed and the results showed that fecal secretory immunoglobulin A (sIgA) titer significantly increased in the probiotic group infants (100). Another study on chicken models showed that a mixture of probiotic *Lactobacillus* spp can enhance IFN-γ gene expression but does not influence antibody production after influenza vaccination (101). Consumption of probiotics containing *Lactobacillus acidophilus; Lactobacillus plantarum; Pediococcus pentosaceus*; *Saccharomyces cerevisiae*; *Bacillus subtilis*, and *Bacillus licheniformis* in broiler chickens resulted in the diminished adverse effect of live vaccine, reduced mortality rate, fecal shedding, and re-isolation of *Salmonella Enteritidis* (SE) from liver, spleen, heart, and cecum against SE vaccine (102). On this subject, oral administration of *L.plantarum GUANKE* (LPG) on mice models acted as a booster for COVID-19 vaccination and boosted >8-fold specific neutralization antibodies in bronchoalveolar lavage (BAL) and >2-fold in serum (103). An *in-vitro* and *in-silico* study showed that *L*.*plantarum* could reduce inflammatory markers such as IFN-α, IFN-β, and IL-6 and block virus replication by interaction with SARS-CoV-2 helicase (104). *L. acidophilus* W37 (LaW37) with long-chain inulin (lcITF) was also used as a probiotic in a study on piglets and increased two-folded vaccine efficacy against *Salmonella Typhimurium* strains (STM) (105).

A pilot study on adults who received the influenza vaccine reported that *L. rhamnosus GG* (LGG) could be an influential adjuvant to improve influenza vaccine immunogenicity (106). LGG also improves T cell responses but not antibody production on human gut microbiota (HGM) transplanted gnotobiotic (Gn) pig model vaccinated with AttHRV (72). However, specific RV antibody production was stimulated in infants who received LGG (107). Another study confirms that the combination of *L*. *acidophilus CRL431* and LGG enhanced IgA and IgM (but not IgG) production after OP vaccination (108).

Other types of probiotics have been studied on this subject as well. For example, *Escherichia coli Nissle* (EcN) 1917 was used to colonize antibiotic-treated and human infant fecal microbiota transplanted Gn piglets and immune response was evaluated to human Rotavirus (HRV). As a result, the humoral and cellular immune responses were enhanced, and EcN biofilm increased the frequencies of systemic memory and IgA + B cells (109, 110). Likewise, the *Lactococcus lactis* strain decreased severity and symptoms in volunteers with Dengue fever (DF) compared to the placebo group, promoted IFN-γ and TGF-β cytokines secretion, and reduced serum IgE and IL-4 cytokine levels in mice models (111, 112). *Bacillus toyonensis* (*B*. *toyonensis*) *BCT-7112* was also enabled to improve the humoral immune response of ewes against the *clostridium perfringens* epsilon toxin (rETX) vaccine and boost higher neutralizing antibody titers (113). *B. toyonensis* and *Saccharomyces boulardii* also successfully boosted antibody production and expression of IFN-γ, IL2, and Bcl6 genes in *Clostridium chauvoei* vaccinated sheep (114). Likewise, *Bacillus velezensis* significantly reduced the pigeon circovirus (PiCV) viral load in the feces and spleen of pigeons and promoted TLR 2&4 expression (115). Fecal microbiome transplantation with *Clostridium butyricum* and *Saccharomyces boulardii* treatment in piglets not only improved plasma concentrations of IL-23, IL-17, IL-22 and specific antibodies against *Mycoplasma hyopneumoniae* (M. hyo) and Porcine Circovirus Type 2 (PCV2), but also decreased the inflammation levels and oxidative stress injury, and improved intestinal barrier function (116).

Although several studies reported a positive effect of *Lactobacillus* on VE, some studies yielded different results. For example, maternal LGG supplementation showed decreased specific antibody responses in tetanus, Haemophilus influenza type b (Hib), and pneumococcal conjugate (PCV7) vaccinated infants (117). Also, probiotic consumption containing *Lactobacillus strains (L. paracasei* and *Lactobacillus casei (L. casei) 431* showed no effects on the immune response to the influenza vaccine but shortened the duration of respiratory symptoms (118). Another study on *L. paracasei* and MoLac-1 (heat-killed) supplemented diet reported the same results, and these probiotics could not boost immune responses after vaccination (119). A recent study also assessed LGG consumption impact on influenza vaccine efficacy in type 1 diabetic (T1D) children and reported no significant improvement in humoral response in the probiotic group (120). In conclusion, although some studies show that probiotics are inefficient in boosting the immune system and increasing vaccine efficacy, most studies demonstrated the positive effects of probiotics on promoting vaccine immunity and protecting the gut barrier simultaneously (Table 1).

**Table 1:** Probiotics' effect on immune responses and vaccine efficacy.

**Probiotic strain**	**Participants**	**Vaccine**	**Effects of probiotics on vaccine response**	**Reference**
*B. longum* *BB536*	Human infants	DTP (diphtheria, pertussis, and tetanus)	An increase in the ratio of IFN-γ/IL-4 secretion cells in the BB536 supplementation group	(92)
*L. paracasei 431*	Human adults	Inactivated trivalent influenza vaccine	No difference in A/H1N1, A/H3N2, and B strain-specific IgG/No difference in A/H1N1, A/H3N2, and B strain-specific IgA levels in saliva / No difference in seroconversion rates 3 w after vaccination	(118)
*L. rhamnosus GG*	Human pregnant women	Combined diphtheria-tetanus-acellular pertussis-Haemophilus influenza type b vaccine	Lower pneumococcal-specific IgG levels/Lower seroconversion rates for pneumococcal serotypes /Lower tetanus toxoid-specific IgG levels/No difference in Hib-specific IgG levels/Higher tolerogenic T regulatory (Treg) responses	(117)
*L. paracasaei* *MoLac-1 (heat-killed)*	Human adults	Inactivated trivalent influenza vaccine	No differences in natural killer cell activity, neutrophil bactericidal or phagocytosis activity/No difference in IgA, IgG, and IgM levels/Higher H3N2 specific IgG levels/No difference in seroconversion rates	(119)
*B. lactis BB-12 / L. paracasei 431*	Human adults	Inactivated trivalent influenza vaccine	An increase in influenza-specific IgG levels/Higher seroconversion rates for IgG/Higher influenza-specific IgA levels in saliva /No differences in NK-cells activity, number of CD4+T-lymphocytes and phagocytosis/No differences in INF-γ, IL-2, and IL-10 levels	(95)
*LGG and inulin*	Human adults	Nasal attenuated trivalent influenza vaccine	Increased seroprotection rate to the H3N2 strain, but not to the H1N1 or B strain	(106)
*B. longum BL999 /L.rhamnosus* *LPR*	Human infants	Hepatitis B Virus (HBV), DTP	An improvement in HepB surface antibody responses in subjects receiving monovalent and a DTPa-HepB combination vaccine at 6 months but not those who received 3 monovalent doses	(93)
*B. bifum / B.infantis/ B.longum/ L.acidophilus*	Human infants	Measles-Mumps-Rubella-Varicella vaccine	Higher overall seroconversion rates/No difference in specific seroconversion rates for rubella, mumps, measles, varicella/No difference in the rate of treatment-related adverse effects between the two groups	(96)
*L. acidophilus* *CRL431/ L. rhamnosus GG*	Human adults	Oral polio vaccine	An increase in poliovirus neutralizing antibody levels/ Increase in poliovirus-specific IgA and IgM levels /No change in poliovirus-specific IgG levels	(108)
*L. casei GG*	Human infants	Oral rotavirus vaccine	Higher number of rotavirus-specific IgM secreting cells/ Higher IgA seroconversion rates /Higher IgM seroconversion rates	(107)
*Escherichia coli Nissle 1917*	Ciprofloxacin (Cipro)-treated Gn piglets colonized with a defined commensal microbiota (DM)	Virulent human rotavirus (HRV)	An increase in the numbers of total immunoglobulin-secreting cells, HRV-specific antibody-secreting cells, activated antibody-forming cells, memory antibody-forming B cells, and naive antibody-forming B cells/ A Decreased in levels of pro-inflammatory but increased levels of immuno-regulatory cytokines and increased frequencies of Toll-like receptor-expressing cells	(109)
*L.rhamnosus GG*	Human gut microbiome transplanted neonatal Gn pig	Attenuated HRV vaccine	Significantly enhancement in HRV-specific IFN-γ producing T cell responses to the AttHRV vaccine. Neither doses of LGG significantly improved the protection rate, HRV-specific IgA and IgG antibody titers in serum, or IgA antibody titers in intestinal contents	(72)
*L. plantarum*	24-Month-old children	-	An increase in fecal sIgA titer /A Significant positive correlation between TGF-β1,TNF-α, and fecal sIgA	(100)
*B. longum + gluco-oligosaccharide*	Human adults	Influenza seasonal vaccine	Significantly higher number of senescent (CD28–CD57+) helper T cells/Significantly higher plasma levels of anti-CMV IgG and a greater tendency for CMV seropositivity/Higher numbers of CD28–CD57+ helper T cells	(94)
*L. plantarum GUANKE (LPG)*	Mice	SARS-CoV-2 vaccine	Enhancement of SARS-CoV-2 neutralization antibodies production/A boost in specific neutralization antibodies >8-fold in bronchoalveolar lavage and >2-fold in sera when LPG was given immediately after SARS-CoV-2 vaccine inoculation /Persistence in T-cell responses	(103)
*Lactococcus lactis strain plasma (LC-Plasma)*	Human adults	Dengue fever (DF)	Significant reduction in the cumulative incidence days of DF-like symptoms/Significantly reduced severity score in the LC-Plasma group	(111)
*Lactobacillus&nbsp;* *plantarum Probio-88*	*In vitro* and *in silico* study	SARS-CoV-2 infection	A significant inhibition in the replication of SARS-CoV-2 and the production of reactive oxygen species (ROS) levels/A significant reduction in inflammatory markers such as IFN-α, IFN-β, and IL-6	(104)
*probiotic Lactobacillus*	Chickens	Herpesvirus of turkeys vaccine	An increase in the expression of major histocompatibility complex (MHC) II on macrophages and B cells in spleen/A decrease in the number of CD4+CD25+ T regulatory	(99)
			cells in the spleen/ higher expression of IFN-α at 21dpi in the spleen/A decrease in the expression of tumor growth factor (TGF)-β4	
*probiotic Escherichia coli Nissle (EcN) 1917*	Malnourished piglet model transplanted with human infant fecal microbiota (HIFM)	HRV vaccine	Increased frequencies of activated plasmacytoid dendritic cells (pDC) and activated conventional dendritic cells (cDC)/increased frequencies of systemic activated and memory antibody-forming B cells and IgA+ B cells in the systemic tissues/Increase in the mean numbers of systemic and intestinal HRV-specific IgA antibody-secreting cells (ASCs), as well as HRV-specific IgA antibody titers in serum and small intestinal contents	(110)
*Bacillus velezensis*	Pigeons	Pigeon circovirus (PiCV)	Significant reduction in the PiCV viral load in the feces and spleen of pigeons/Up-regulation in Interferon-gamma (IFN-γ), myxovirus resistance 1 (Mx1), signal transducers and activators of transcription 1 (STAT1), toll-like receptor 2 (TLR2) and 4 (TLR4)gene expression	(115)
*Lactococcus lactis NZ1330*	BALB/c Mouse Model	Allergy to Amaranthus retroflexus pollens	Significantly reduction in the serum IgE level/Best performance in terms of improving allergies to Th1 and Treg responses	(112)
*L.acidophilus; L.plantarum; Pediococcus pentosaceus; Saccharomyces cerevisiae; B.subtilis; B.licheniformis*	Broiler chickens	Salmonella Enteritidis (SE) vaccine	Diminished the negative effect of live vaccine growth performance/reduced mortality rate, fecal shedding, and re-isolation of SE from liver, spleen, heart, and cecum	(102)
*long-chain inulin (lcITF) and L.acidophilus W37 (LaW37)*	Piglets	Salmonella Typhimurium strains (STM)	Enhanced vaccination efficacy by 2-fold /Higher relative abundance of Prevotellaceae and lower relative abundance of Lactobacillaceae in feces/Increased the relative abundance of fecal lactobacilli was correlated with higher fecal consistency	(105)
*fecal microbiome+ Clostridium butyricum and Saccharomyces boulardii*	Gpiglets	-	Increased the plasma concentrations of IL-23, IL-17, and IL-22, as well as the plasma levels of anti-M.hyo and anti-PCV2 antibodies/ Decreases in inflammation levels and oxidative stress injury, and improvement of intestinal barrier function	(116)
*L.rhamnosus GG (LGG)*	Patients with type 1 diabetes	Betapropiolactone- whole inactivated virus	Reduction in the inflammatory responses (i.e., IFN-γ, IL17A, IL-17F, IL-6, and TNF-α)/Significantly	(120)
			production of IL-17F prior to and after (90 ± 7 days later) vaccination	
*B. toyonensis BCT-7112T*	Ewes of the Corriedale sheep	Recombinant Clostridium perfringens epsilon toxin (rETX)	Higher neutralizing antibody titers/An increase in serum levels for total IgG anti-rETX/Increase IgG isotypes IgG1 and IgG2 /Higher cytokine mRNA transcription levels for IL-2, IFN-γ, and transcription factor Bcl6	(113)
*B. toyonensis and Saccharomyces boulardii*	Sheep	Clostridium chauvoei vaccine	Significantly higher specific IgG, IgG1, and IgG2 titers/Approximately 24- and 14-fold increases in total IgG levels/ Increased mRNA transcription levels of the IFN-γ, IL2, and Bcl6 genes	(114)

## Probiotic-based vaccines

**Figure 1:**
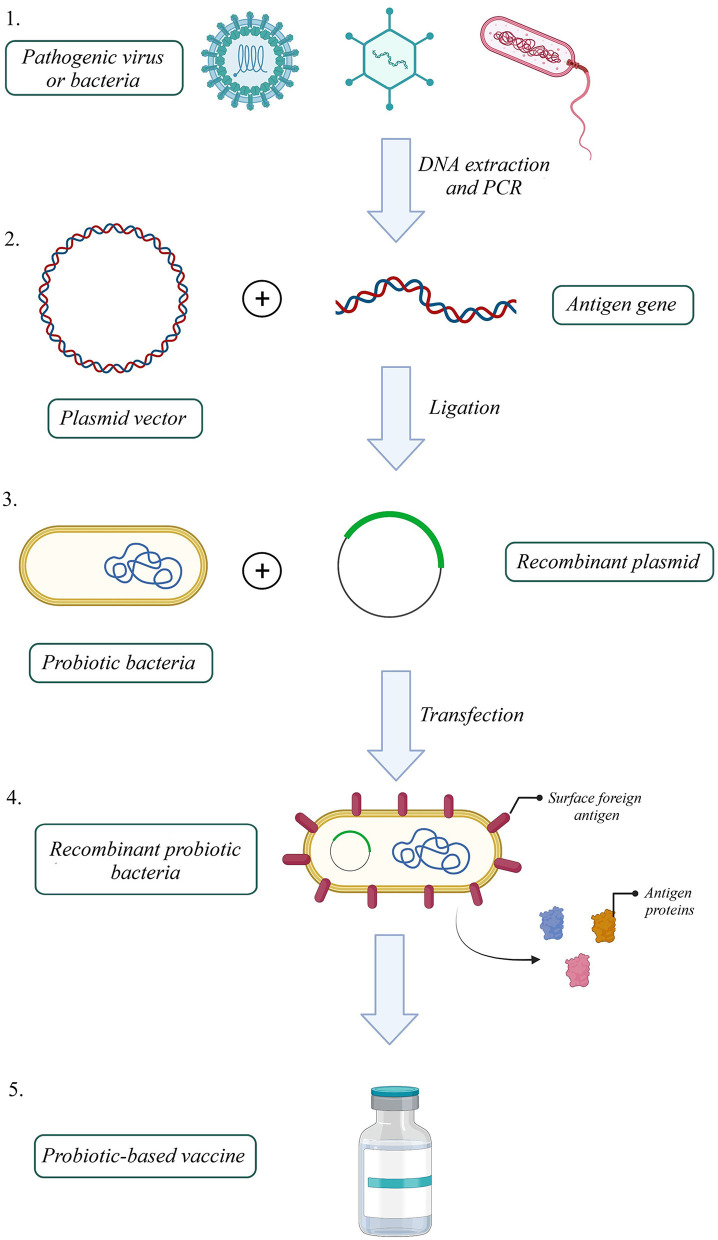
How to build a probiotic-based vaccine: 1. Extract the antigen gene from the pathogen, 2. Amplify the gene by polymerase chain reaction (PCR), 3. Build a recombinant expression plasmid by ligating antigen gene into a proper plasmid, 4. Transfect recombinant plasmid into a probiotic host, 5. Select successfully transfected recombinant probiotic bacteria 6. Probiotic-based oral vaccines could be manufactured with a recombinant probiotic host expressing the pathogenic antigen (Created with BioRender.com).

One efficient way to increase VE, produce a better immune response to an antigen, and reduce attenuated vaccine risk is to utilize recombinant antigens in gut microbiota vectors. Based on this idea, several probiotic-based vaccines were developed (Figure 1). For instance, the recombinant *Streptococcus gordonii* RJM4 vector has been used to express the N-terminal fragment of the S1 subunit of pertussis toxin (PT) as a SpaP/S1 fusion protein in mice. SIgA in saliva and IgG were detected, and long-term oral colonization and maintenance of recombinant protein were observed in these animal models (121). The B subunit of the heat-labile toxin (LTB) was one of the antigen targets that colonized *Bacillus subtilis* (B. *subtilis*) with episomal expression systems. Vaccinated mice with engineered *B. subtilis via* the oral route could be recognized and neutralize the native toxin, produced by enterotoxigenic *Escherichia coli* (ETEC) strains *in vitro* (122). B. *subtilis* was also used as a shuttle for *Clonorchis sinensis* antigen. Compared with control groups, the results indicated that the vaccinated group could induce humoral and cellular immune responses successfully (123). Furthermore, another vaccine against ETEC strains, the probiotic *E. coli Nissle* 1917 (EcN) was used to express Stx B-subunits, OspA, and OspG protein antigens. This system could elicit hormonal responses but could not trigger selective T-cell responses against selected antigens (124). On the other hand, *EcN 1917* expressing heterologous F4 or F4 and F18 fimbriae of ETEC improved anti-F4 and both anti-F4 and anti-F18 IgG immune responses (125).

*Lactococcus lactis* is a commonly used food-grade probiotic. To develop a vaccine against *Helicobacter pylori, L. lactis* expressing Helicobacter pylori urease subunit B (UreB) was used and results demonstrated that orally vaccinated mice elicited significant humoral immunity against gastric Helicobacter infection (126). Tang et al., designed a recombinant *L. lactis* expressing TGEV spike glycoprotein. Results on mice revealed induction of local mucosal immune responses and IgG and IgA antibodies production against TGEV spike glycoprotein (127). On this subject, *L. lactis* PppA (LPA+) recombinant strain containing pneumococcal protective protein A (LPA) in oral immunized mice showed mucosal and systemic antibody production against different serotypes of *Streptococcus pneumonia* (128). *L. lactis* was likewise used to deliver rotavirus spike-protein subunit VP8 in the mouse model. The serum of animals that received *L*. *lactis* with cell wall-anchored RV VP8 antigen could inhibit viral infection *in vitro* by 100% and vaccinated mice developed significant levels of intestinal IgA antibodies *in vivo* (129). The oral vaccine with *L*. *Lactis* expressing a recombinant fusion protein of M1 and HA2 proteins derived from the H9N2 virus successfully induces protective mucosal and systemic immunity in eighty 1-day-old chickens (130). Mohseni et al. employed *L. lactis* as a vector for expressing the codon-optimized human papillomavirus (HPV) - 16 E7 oncogenes, and it showed cytotoxic T lymphocytes (CTL), and humoral responses after vaccination in healthy women volunteers with this probiotic-based vaccine (131). Similarly, another study on *L*. *lactis* expressing HPV codon-optimized E6 protein reported induction of humoral and cellular immunity and significantly increased intestinal mucosal lymphocytes, splenocytes, and vaginal lymphocytes in the vaccinated group compared to controls (132).

*Lactobacillus casei* strains are known for their immune stimulatory effect and have been used as probiotics for many years. A genetically engineered *L. casei* oral vaccine expressing dendritic cell (DC)-targeting peptide for Porcine epidemic diarrhea (PED) resulted in significantly elevated levels of anti-PEDV specific IgG and IgA antibody responses in mice and piglets (133, 134). Yoon et al. expressed poly-glutamic acid synthetase A (pgsA) protein from HPV-16 L2 in *L*. *casei*, and interestingly, L2-specific antibodies had cross-neutralizing activity against diverse HPV types in the mouse model (135). Recombinant *L. casei* was also used for immunizing piglets against TGEV. As a result, solid cellular response, switching from Th1 to Th2-based immune responses, and IL-17 expression in systemic and mucosal immunity was reported (136). In another study, α, ε, β1, and β2 toxoids of *Clostridium perfringens* expressed in *L. casei* ATCC 393 vector and elevated the levels of antigen-specific mucosa sIgA and sera IgG antibodies with exotoxin-neutralizing activity were seen in rabbit models (137). A different study used this probiotic expressing the VP2 protein of infectious pancreatic necrosis virus (IPNV) and reported induction of local mucosal and systemic immune responses in rainbow trout juveniles (138).

Other strains of *lactobacillus* are used in this technique as well. Oral recombinant *Lactobacillus* vaccine containing VP7 antigen of porcine rotavirus (PRV) showed stimulation in the differentiation of dendritic cells (DCs) in Peyer's patches (PPs) significantly, increased serum levels of IL-4 and IFN-γ and production of B220+ B cells in mesenteric lymph nodes (MLNs). Also, it increased the titer levels of the VP7-specific antibodies in mice models (139). Recombinant *L. Plantarum* expressing H9N2 avian influenza virus used for specific pathogen-free (SPF) 3-week-old chickens and could elicit humoral and cellular immunity (140). Shi et al. showed excessive serum titers of hemagglutination-inhibition (HI) antibodies in mice, and robust T cell immune responses in both mouse and chicken H9N2 vaccinated models by Recombinant *L. Plantarum* (141). *L. Plantarum* NC8, expressing oral rabies vaccine G protein fused with a DC-targeting peptide (DCpep), resulted in more functional maturation of DCs and a strong Th1-biased immune response in mice (142). A recent study utilized *L*. *Plantarum* for developing SARS-CoV-2 food-grade oral vaccine. The results indicated that the spike gene could be efficiently expressed on the surface of recombinant *L*. *Plantarum* and displayed high antigenicity (143). As a novel approach for vaccination against SARS-Cov2, *L. plantarum* strain expressing the SARS-CoV-2 spike protein was used, and high yields for S protein were obtained in an engineered probiotic group *in vitro* (143). In murine models, *Lactobacillus pentosus* expressing D antigenic site of spike glycoprotein transmissible gastroenteritis coronavirus (TGEV) could induce IgG and sIgA against this virus (144). Recombinant *Lactobacillus rhamnosus* that contains Koi herpesvirus (KHV) ORF81 protein in vaccinated fish was also successfully generated antigen-specific IgM with KHV-neutralizing activity (145). Another study used *Lactobacillus acidophilus* vector with the membrane-proximal external region from HIV-1 (MPER) and secreted interleukin-1ß (IL-1ß) or expressed the surface flagellin subunit C (FliC) as adjuvants, and reported as an improved vaccine efficacy and immune response against HIV-1 in mice (146). These studies demonstrated that probiotics have a potential role in acting as a shuttle for recombinant oral vaccines and successfully promoting the immune system against pathogens, and improving intestinal condition simultaneously.

## Future perspective

There is no doubt that gut microbiota significantly impacts human metabolism and the immune system. Even further, some scientists consider gut microbiota as an endocrine organ in the human body. Probiotics are part of gut microbiota that have health benefits and promote immune responses. Based on the impact of gut mucosal immunity in the humoral immune response to vaccination, using probiotics as an immune booster next to oral vaccines can lead to better immunity, and probiotic-based recombinant vaccines promise a better generation of recombinant vaccines. Although a few human studies were performed on this subject, probiotics and probiotic-based recombinant vaccines' efficacy on immunity against pathogens is promising. Such a new oral vaccine against SARS-CoV-2 infection was developed by Symvivo Corporation (a Vancouver-based Biotech Company) using *Bifidobacteria longum*, for expressing spike protein (named bacTRL-Spike), and it is under investigation in phase 1 clinical trials (NCT04334980). However, more studies need to be performed to detect the effectiveness of probiotics and engineered probiotic vaccines in clinical trials and investigate their role in human immunological pathways to ensure their safety and durable immunity.

## Author contributions

NK: literature search, writing, and drawing of figures. AD and SB: literature search. All authors contributed to the article and approved the submitted version.

## Conflict of interest

The authors declare that the research was conducted in the absence of any commercial or financial relationships that could be construed as a potential conflict of interest.

## Publisher's note

All claims expressed in this article are solely those of the authors and do not necessarily represent those of their affiliated organizations, or those of the publisher, the editors and the reviewers. Any product that may be evaluated in this article, or claim that may be made by its manufacturer, is not guaranteed or endorsed by the publisher.
